# Clinical Efficacy of Treating Endometrial Cancer with Xiaoaiping Tablets under Comprehensive Nursing Intervention and Their Effect on Quality of Life

**DOI:** 10.1155/2021/2035361

**Published:** 2021-10-13

**Authors:** Juan Wang, Qingyan Liu, Jianping Li, Dan Nie, Daiying Zhang

**Affiliations:** ^1^Department of Operating Room, Affiliated Hospital of Southwest Medical University, Luzhou, Sichuan 646000, China; ^2^Department of Gynaecology, Affiliated Hospital of Southwest Medical University, Luzhou, Sichuan 646000, China

## Abstract

**Objective:**

To explore the clinical efficacy of treating endometrial cancer with Xiaoaiping tablets under comprehensive nursing intervention and their effect on quality of life.

**Methods:**

The clinical data of 120 endometrial cancer patients treated at the *Affiliated Hospital of Southwest Medical University* from February 2019 to February 2020 were retrospectively analyzed, and the patients were split into the experimental group and the control group according to their admission order, with 60 cases each. Conventional treatment and Xiaoaiping tablet regimen were received by all patients, those in the control group accepted the general nursing, and those in the experimental group accepted the comprehensive nursing intervention for 12 months, so as to compare their clinical efficacy, quality of life (Functional Assessment of Cancer Therapy, FACT), negative emotion scores (Hospital Anxiety and Depression Scale, HAD), and Medical Coping Modes Questionnaire (MCMQ) scores between the two groups.

**Results:**

No statistical differences in the patients' general information between the two groups were observed (*P* > 0.05); compared with the control group after nursing, the experimental group obtained a significantly higher objective remission rate (80.0%), significantly higher disease control rate (90.0%) (*P* < 0.05), significantly better QOL (*P* < 0.001), significantly lower negative emotion scores (*P* < 0.001), and significantly better MCMQ scores (*P* < 0.001).

**Conclusion:**

Adopting Xiaoaiping tablets under comprehensive nursing intervention can improve the negative emotions of patients with endometrial cancer, enhance their confidence in medical treatment, present better efficacy, and obviously promote their QOL. Therefore, comprehensive nursing intervention should be promoted and applied in practice.

## 1. Introduction

Endometrial cancer is an epithelial malignancy that occurs in the endometrium, with an incidence accounting for 7% in female malignancies and 25%–30% in genital malignancies [1, 2]. The number of new patients with endometrial cancer per year in China is around 200,000 [3], whose risk level is second only to that of cervical cancer. With the rising incidence of endometrial cancer, clinical research on its treatment measures is also continuously deepening. At the present stage, surgery and chemotherapy are mainly adopted to treat the disease. To be specific, early-stage endometrial cancer is mainly treated by surgery, chemoradiotherapy and other treatment measures can be performed jointly after surgery according to the pathological stage of patients, and comprehensive treatment is conducted for patients at the advanced stage [4, 5]. Generally, surgery supplemented with chemoradiotherapy is the conventional treatment for malignant tumors, but endometrial cancer belongs to reproductive system tumors, which has a significant impact on female physical and mental health, so the treatment effect of patients is subject to many factors such as physiological conditions and mental states. Also, some of the middle-aged and elderly patients with underlying diseases have a particularly poor quality of life (QOL) [6, 7], low body tolerance, and serious hostile and negative mentality, which are not conducive to obtaining the best surgical treatment effect. To solve the problems such as poor treatment tolerance of endometrial cancer patients, some scholars treated such patients with Xiaoaiping tablets and found that the drug can significantly improve the body immunity of patients, thereby reducing the toxic side effects caused by chemotherapy [8], and that the medication will not increase the pain sensation of patients and presents exact efficacy. However, endometrial cancer can cause strong mental stimulation, so patients are prone to mental system dysfunction, and some even have suicidal tendencies [9]. Such negative coping style seriously affects the psychological defense mechanism of patients and significantly reduces their treatment compliance. Therefore, jointly adopting routine nursing on the basis of Xiaoaiping tablet treatment cannot improve the patients' psychological state, and more comprehensive nursing intervention measures should be adopted jointly.

Comprehensive nursing intervention is an all-round and high-quality nursing model capable of rising nursing intervention for cancer from traditional adjuvant therapy to humanities care so that patients can obtain more targeted quality nursing during treatment, which in turn changes their medical coping attitudes and improves treatment compliance [10]. In previous literature, no study has applied comprehensive nursing in endometrial cancer patients treated with Xiaoaiping tablets. Based on this, the actual effect of the comprehensive nursing intervention was explored herein, with the results reported as follows.

## 2. Data and Methods

### 2.1. Study Design

The study was a retrospective study and conducted at the *Affiliated Hospital of Southwest Medical University* from February 2019 to February 2020 to explore the clinical efficacy of treating endometrial cancer with Xiaoaiping tablets under comprehensive nursing intervention and their effect on QOL. This study was a double-blind study, meaning that neither the study subjects nor the researchers were aware of trial grouping, and the study designer was responsible for arranging and controlling the full trial.

### 2.2. Recruitment of Study Objects

The clinical data of patients with endometrial cancer treated at the *Affiliated Hospital of Southwest Medical University* from February 2019 to February 2020 were retrospectively analyzed, and the patients were included and excluded according to the following criteria. Inclusion criteria included the following: (1) the patients were diagnosed with endometrial cancer after B-mode ultrasound, hysteroscope, and magnetic resonance imaging (MRI) examinations and met the diagnosis criteria in the fourth version of *Guidance for Diagnosis and Treatment of Endometrial Cancer* [11] published in 2018; (2) the patients had measurable lesions; namely, the diameter of lesions was not less than 20 mm under routine imaging examination; (3) the patients were under the age of 80 years; (4) the estimated survival of the patients was over 3 months; (5) the patients' KPS scores were not less than 65 points [12]; and (6) throughout the treatment, no death, transfer, or treatment discontinued occurred to the patients. Exclusion criteria included the following: (1) the patients could not communicate with others due to hearing disorders, language disorders, unconsciousness, mental diseases, or other factors; (2) the patients quit the treatment, died, changed the treatment regimen, or were found missing in follow-up visit; (3) the patients suffered from other severe organic diseases; (4) tumor distant metastasis occurred; and (5) the patients were alcohol dependent.

### 2.3. Steps

A total of 120 patients were included in the study and equally divided into the experimental group and the control group according to their admission order, with 60 cases each. On the day that the patients agreed to join the study, the study team collected the sociodemographic data and clinical performance data and distributed the questionnaires for all patients to fill in under unified guidance, the nursing personnel made no implications except for explaining the questionnaires, and after filling, the questionnaires were taken back and verified for completeness; after the end of nursing, the imaging examination was performed to the patients, and the questionnaires were distributed again and taken back after filling for completeness verification. In case of any omissions, the patients were encouraged to complete the questionnaires on the basis of no violation of the voluntary principle to ensure integrity and authenticity.

### 2.4. Moral Consideration

The study met the principle of the World Medical Association Declaration of Helsinki [13] and was approved by the Ethics Committee of the Affiliated Hospital of Southwest Medical University. After the patients were recruited, the study team explained the purpose, meaning, content, and confidentiality of the study to them and asked them to sign the informed consent.

### 2.5. Criteria of Quitting the Experiment

For patients who had one of the following situations and were judged unsuitable for continuously accepting the experiment by the study team, their case records were reserved but not used for data analysis: (1) those experienced adverse events or severe adverse events; (2) progression of disease and so on occurred during the experiment; (3) subjects developed some serious comorbidities or complications; and (4) subjects were unwilling to continue the clinical trial and proposed the demand to quit.

### 2.6. Methods

Conventional treatment combined with Xiaoaiping tablets (manufactured: Jilin Jichun Pharmaceutical Co., Ltd., NMPA approval no. Z20063564) was performed on patients in both groups. The patients orally took 8 Xiaoaiping tablets (0.3 g per tablet) three times daily for a total of 4 courses, with 3 weeks as 1 course.

The patients in the control group accepted the general nursing as follows: (1) The nursing personnel paid close attention to related indicators of the patients and dynamically monitored the changes in their vital signs. (2) The nursing personnel guided the patients to take drugs according to medical advice and gave diet guidance and health guidance to the patients, thereby promoting their treatment compliance.

The comprehensive nursing intervention was conducted on patients in the experimental group as follows: (1) The nursing personnel carefully read the patients' basic information, prepared targeted nursing plans with individual differences according to the patients' educational degree, condition, dietary preference, and so on, made the nursing plans more scientific and professional by evidence-based nursing, and adjusted the plans at any times according to the feedback from the patients during nursing, thus ensuring that the patients accepted high-quality comprehensive nursing. (2) Psychological nursing: the nursing personnel accepted the training by a professional psychological consultant to promote the accuracy of psychological nursing and, in particular, made psychological nursing plans for specific psychological situations of patients with endometrial cancer, mainly including the following aspects: ① The nursing personnel had one-on-one communication with the patients daily, informed the patients about the influence of their psychological status on treatment, increased their degree of recognition for psychological intervention, and at the same time evaluated their mental status and gave emotional support. ② For patients with poor psychological status, the nursing personnel helped them in relieving their stress and negative emotions such as anxiety and hostility by the means of cognitive therapy and imagery communication. ③ In case that patients presented serious negative emotions and even mental diseases or somatic symptoms, relevant treatment should be intervened upon immediately. (3) Health education: ① The nursing personnel carried out health education to patients according to their educational degree, the senior medical staff introduced the basic knowledge of the disease through lectures, distributed corresponding health education handbooks to the patients after lecture, and informed the patients about the characteristics of endometrial cancer, so that the patients understood that the disease is progressing slowly but often has a good prognosis and then enhanced their confidence in treatment. ② The nursing personnel taught the family members of the patients at the same time, informed them that endometrial cancer will not affect their sexual life or accelerate the aging of women, and advised them to promote their degree of attention to the patients and be more caring and encouraging, and for those with poorer family nursing ability, the nursing personnel explained the nursing methods to them privately. ③ The nursing personnel could organize patients' exchange meeting and arrange the patients who obtained the ideal treatment effect to be the speaker, thereby exerting the peer effect and helping the patients in correctly recognizing cancer and accepting the treatment and nursing regimen. (4) Other nursing measures: ① The patients with endometrial cancer often complained of pain, so the nursing personnel needed to administer them with ibuprofen and other drugs according to the doctor's advice, closely monitor their drug reaction, and if necessary adopt the psychological adjustment method for distraction. ② Endometrial patients should eat foods that contain rich protein, so the nursing personnel prepared healthy recipe according to their dietary habits and advised them about eating fewer foods with high cholesterol and calories and keeping off spicy food. ③ The cancer patients had different degrees of sleep disorders, so the nursing personnel should keep the hospitalization environment clean and tidy and tell the patients to take a hot bath and massage before sleep to improve their sleep quality.

### 2.7. Observation Criteria

1. General information: the general information extract form was established by the patient, covering the inpatient number, name, age, body weight, BMI, cancer stage, pathological stage, pathological type, number of lesions, KPS scores, marital status, and educational degree.
2. Clinical efficacy: one month after the end of medication, patients in both groups accepted the pelvis CT scan and MRI examination, and their short-term efficacy was evaluated by the Response Evaluation Criteria in Solid Tumors (RECICT) [14] and classified as complete response (CR, complete endometrial retraction, interstitial decidua degeneration, and no endometrial hyperplasia or cancer focus for more than one month), partial response (PR, reduced level of endometrial lesions or residual cancer focus, accompanied by gland degeneration and atrophy for over one month), stable disease (SD, no changes in endometrium, residual cancer focus, and no degeneration and atrophy seen in endometrium), and progressive disease (PD, clear muscular invasion or extrauterus lesions). The objective remission rate (ORR) = CR + PR, and the disease control rate (DCR) = CR + PR + SD.
3. QOL: the patients' QOL before and after nursing was evaluated based on the Functional Assessment of Cancer Therapy-General (FACT-G) [15], including physical well-being (PWB), social/familial well-being (SWB), emotional well-being (EWB), and functional well-being (FWB), and a total of 27 evaluation items. The scores of PWB, SWB, and FWB ranged from 0 to 28 points, and the score of EWB ranged from 0 to 24 points, with 0 points indicating the best score for QOL.
4. Negative emotion scores: the patients' negative emotions before and after nursing were evaluated based on the Hospital Anxiety and Depression Scale (HAD) [16], which was divided into the scales for anxiety and depression (HAD-A and HAD-D). With the credibility and validity that have been proved in domestic literature, HAD can be used to evaluate the negative emotions in patients with endometrial cancer in China. The scores of both scales ranged from 0 to 15 points, with 0 points indicating the best score of no anxiety and higher scores indicating more serious anxiety and depression.
5. Medical Coping Modes Questionnaire (MCMQ) scores: the patients' medical coping attitude before and after nursing was evaluated by the MSMQ scores [17], which was mainly used for patients with fatal diseases or nonfatal diseases. With the credibility and validity that have been proved in domestic literature, MSMQ can be used to evaluate the medical coping attitude of patients with endometrial cancer in China. MSMQ covered three coping strategies, that is, confrontation, avoidance, and acceptance-resignation, of which the confrontation dimension was in direct ratio with the medical coping, whereas the dimensions of avoidance and acceptance-resignation were in inverse ratio with the medical coping. Each strategy contained different items, and a total of 20 items were included in the Chinese version.

### 2.8. Statistical Processing

In this study, the data processing software was SPSS20.0, the picture drawing software was GraphPad Prism 7 (GraphPad Software, San Diego, USA), items included were enumeration data and measurement data, methods used were *X*^2^ test and *t*-test, and differences were considered statistically significant at *P* < 0.05.

## 3. Results

### 3.1. Comparison of Patients' General Information

No statistical differences in patients' general information between the two groups were observed (*P* > 0.05); see Table 1.

### 3.2. Comparison of Patients' Clinical Efficacy

The ORR and DCR of the experimental group were significantly higher than those of the control group (*P* < 0.05) (see Table 2).

### 3.3. Comparison of Patients' QOL

**Figure 1:**
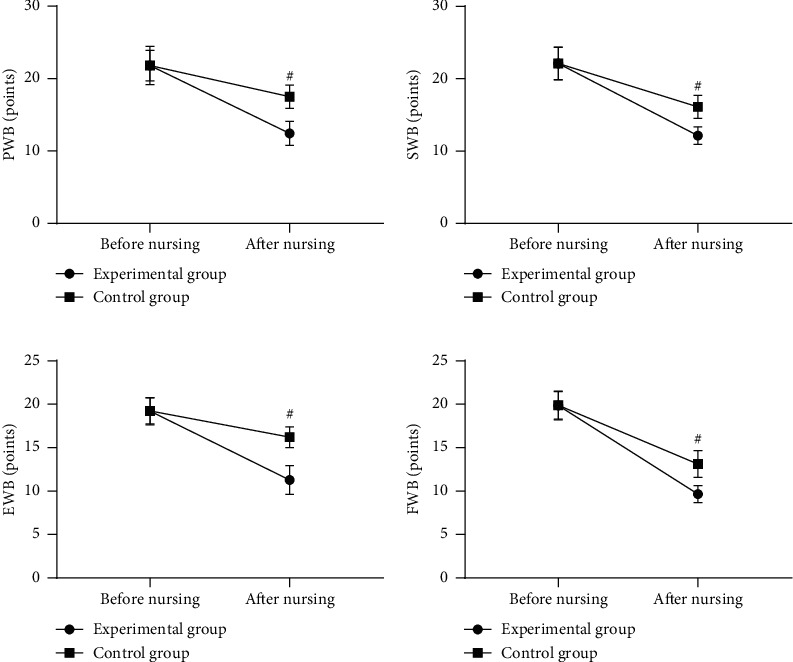
Comparison of patients' QOL ($\bar{x}\pm s$, points). *Note*. The horizontal axes from left to right indicate before and after nursing, the lines with dots indicate the experimental group, the lines with blocks indicate the control group; and # indicates *P* < 0.001.

Compared with the control group after nursing, the patients' QOL was significantly better in the experimental group (*P* < 0.001) (see Figure 1).

Figure 1(a) shows the PWB scores. Before nursing, the PWB scores of the experimental group and the control group were not statistically different (21.80 ± 2.12 versus 21.81 ± 2.65, *P* > 0.05), and after nursing, the PWB scores were significantly lower in the experimental group than in the control group (12.45 ± 1.65 versus 17.52 ± 1.60, *P* < 0.001).

Figure 1(b) shows the SWB scores. Before nursing, the SWB scores of the experimental group and the control group were not statistically different (22.10 ± 2.23 versus 22.12 ± 2.24, *P* > 0.05), and after nursing, the SWB scores were significantly lower in the experimental group than in the control group (12.14 ± 1.20 versus 16.11 ± 1.58, *P* < 0.001).

Figure 1(c) shows the EWB scores. Before nursing, the EWB scores of the experimental group and the control group were not statistically different (19.21 ± 1.58 versus 19.23 ± 1.50, *P* > 0.05), and after nursing, the EWB scores were significantly lower in the experimental group than in the control group (11.28 ± 1.65 versus 16.21 ± 1.20, *P* < 0.001).

Figure 1(d) shows the FWB scores. Before nursing, the FWB scores of the experimental group and the control group were not statistically different (19.87 ± 1.65 versus 19.88 ± 1.57, *P* > 0.05), and after nursing, the FWB scores were significantly lower in the experimental group than in the control group (9.65 ± 0.98 versus 13.12 ± 1.54, *P* < 0.001).

### 3.4. Comparison of Patients' Negative Emotion Scores

**Figure 2:**
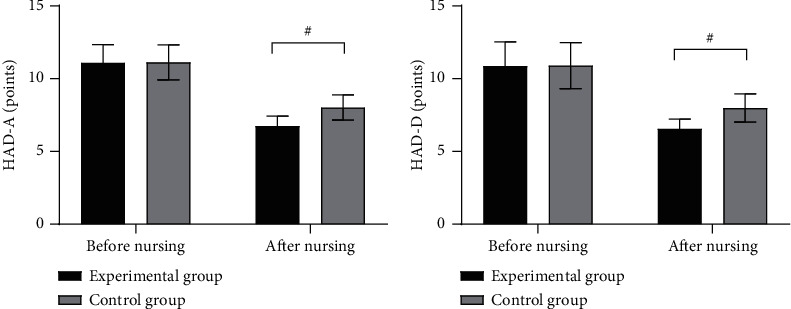
Comparison of patients' negative emotion scores ($\bar{x}\pm s$, points). *Note*. The horizontal axes from left to right indicate before and after nursing, the black areas indicate the experimental group, the gray areas indicate the control group, and # indicates *P* < 0.001.

Compared with the control group after nursing, the negative emotion scores were significantly lower in the experimental group (*P* < 0.001) (see Figure 2).

Figure 2(a) shows the HAD-A scores. Before nursing, the HAD-A scores of the experimental group and the control group were not statistically different (11.10 ± 1.23 versus 11.12 ± 1.20, *P* > 0.05), and after nursing, the HAD-A scores were significantly lower in the experimental group than in the control group (6.74 ± 0.68 versus 8.01 ± 0.87, *P* < 0.001).

Figure 2(b) shows the HAD-D scores. Before nursing, the HAD-D scores of the experimental group and the control group were not statistically different (10.87 ± 1.65 versus 10.89 ± 1.58, *P* > 0.05), and after nursing, the HAD-D scores were significantly lower in the experimental group than in the control group (6.54 ± 0.68 versus 7.98 ± 0.98, *P* < 0.001).

### 3.5. Comparison of Patients' MCMQ Scores

**Figure 3:**
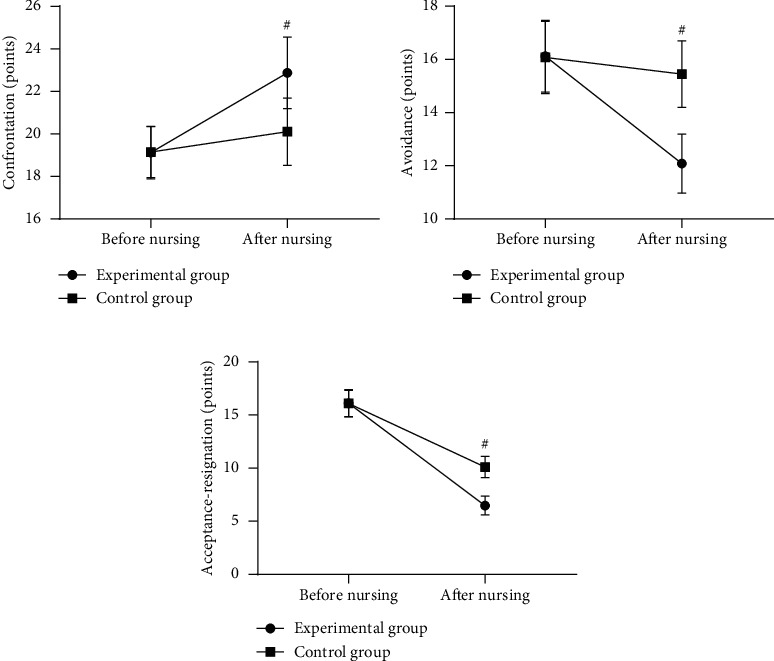
Comparison of patients' MCMQ scores ($\bar{x}\pm s$, points). *Note.* The horizontal axes from left to right indicate before and after nursing, the lines with dots indicate the experimental group, the lines with blocks indicate the control group; and # indicates *P* < 0.001.

Compared with the control group after nursing, the MCMQ scores were significantly better in the experimental group (*P* < 0.001) (see Figure 3).

Figure 3(a) shows the confrontation scores. Before nursing, the confrontation scores of the experimental group and the control group were not statistically different (19.12 ± 1.23 versus 19.15 ± 1.20, *P* > 0.05), and after nursing, the confrontation scores were significantly higher in the experimental group than in the control group (22.87 ± 1.68 versus 20.10 ± 1.58, *P* < 0.001).

Figure 3(b) shows the avoidance scores. Before nursing, the avoidance scores of the experimental group and the control group were not statistically different (16.12 ± 1.35 versus 16.08 ± 1.36, *P* > 0.05), and after nursing, the avoidance scores were significantly lower in the experimental group than in the control group (12.08 ± 1.11 versus 15.45 ± 1.25, *P* < 0.001).

Figure 3(c) shows the acceptance-resignation scores. Before nursing, the acceptance-resignation scores of the experimental group and the control group were not statistically different (16.11 ± 1.28 versus 16.09 ± 1.23, *P* > 0.05), and after nursing, the acceptance-resignation scores were significantly lower in the experimental group than in the control group (6.48 ± 0.87 versus 10.11 ± 1.00, *P* < 0.001).

## 4. Discussion

In China, endometrial cancer is the second leading female reproductive malignancy with an incidence second only to cervical cancer. With the popularity of xenoestrogen in recent years, the incidence of endometrial cancer shows an obvious upward trend, so deepening the research on its therapeutic measures is an urgent task. At the present stage, surgery and chemotherapy are mainly adopted to treat endometrial cancer, and surgical treatment is curative for most patients, achieving a 5-year overall survival rate of 98% and a disease-free survival rate of over 90% [18]. But surgical treatment alone does not improve the 5-year survival for patients with combined core muscles and lymphatic vessel clearance invasion and those at the advanced stage when diagnosed, so chemotherapy regimens are recommended according to relevant guidelines to treat such patients [19]. Chemotherapy can prolong the survival time of patients, but this treatment modality has significant toxic effects. In addition, it often works poorly in patients with endometrial cancer, because such disease often occurs in perimenopausal and postmenopausal women who have poor tolerance, which is not conducive to exerting the effect of chemotherapy. To assist in the chemoradiotherapy and postoperative treatment of patients with endometrial cancer, some scholars administered 134 endometrial cancer patients with Xiaoaiping tablets and found that those in the observation group after administration had significantly improved immune function and achieved a 2-year survival rate of 52.8% [20], which was significantly higher than the statistical result of the international Gynecologic Oncology Group in 2010, suggesting that Xiaoaiping tablets could significantly prolong the survival time of patients. However, endometrial cancer is closely related to female reproductive organs and will cause the lack of physiological structure, seriously affecting patients' social role function, so patients often present negative emotions such as anxiety and depression after diagnosis. In particular, after surgical treatment and chemotherapy, patients suffer from organic trauma again, resulting in significantly reduced treatment compliance and failure to cooperate with the implementation of adjuvant treatment [21, 22]. Therefore, it is extremely important to adopt comprehensive nursing intervention to enhance the therapeutic effects of patients.

Comprehensive nursing intervention refers to integrating humanities care throughout nursing under the guidance of nursing diagnosis and based on the theory of special science, which can provide patients with high-quality and all-round nursing care, fully motivate patients' potential of rehabilitation, and improve patients' psychological and mental status and physical function [23]. In this study, the social psychology-based comprehensive nursing intervention was adopted, and the negative emotion scores after nursing were significantly lower in the experimental group than in the control group (*P* < 0.001), indicating that the psychological nursing in this model could relieve anxiety and depression in patients and improve their psychological function. In the study by scholars David et al., it was shown that psychological intervention could reduce the levels of cortisol and adrenaline in endometrial cancer patients with diabetes, demonstrating that psychological intervention was an important measure to reduce the stress reaction of patients [24]. Stress reaction will lead to various kinds of somatic symptoms in patients and then cause potential organ injury during treatment, so controlling stress reaction is beneficial to protect the body tolerance of endometrial cancer patients and avoid posttraumatic stress disorder (PTSD).

In addition, the psychological and mental status of endometrial cancer patients are closely related to their treatment compliance, and according to American Association for Cancer Research, patients' medical coping style, social support degree, and QOL all affect their will to live. With a reduced sense of survival, patients present poorer treatment compliance and refuse to cooperate with clinical treatment [25]. After the comprehensive nursing intervention, patients in the experimental group had a more optimal level of health knowledge, more scientific daily living habits, and increased social support, and therefore they obtained significantly better MCMQ scores (*P* < 0.001) and better QOL. Hence, comprehensive nursing intervention can improve not only the patients' subjective treatment willingness and treatment compliance but also their objective organism conditions, so the ORR and DCR in the experimental group were significantly higher than those in the control group (*P* > 0.05), indicating that comprehensive nursing intervention incorporated both the inner core of humanism and the strictness of evidence-based nursing and could effectively improve the treatment outcomes of patients with endometrial cancer.

In conclusion, adopting Xiaoaiping tablets under comprehensive nursing intervention can improve the negative emotions of patients with endometrial cancer and enhance the treatment effect of Xiaoaiping tablets, presenting a good clinical application value.

## Figures and Tables

**Table 1:** Comparison of patients' general information.

Group	Experimental (*n* = 60)	Control (*n* = 60)	*X* ^2^/*t*	*P*
Age (years)				
Range	42–76	40–74		
Mean age	50.98 ± 3.65	51.23 ± 3.20	0.399	0.691
Mean body weight (kg)	55.10 ± 2.65	55.14 ± 2.48	0.085	0.932
BMI (kg/m^2^)	22.15 ± 1.20	22.25 ± 1.43	0.415	0.679
Tumor stage				
G1	32	30	0.134	0.715
G2	18	20	0.154	0.695
G3	10	10	0.000	1.000
Pathological stage				
I	24	25	0.035	0.853
II	16	15	0.044	0.835
III	12	13	0.051	0.822
IV	8	7	0.076	0.783
Pathological type				
Adenocarcinoma	36	35	0.035	0.853
Adenosquamous carcinoma	10	8	0.261	0.609
Clear cell carcinoma	8	10	0.261	0.609
Papillary adenocarcinoma	6	7	0.086	0.769
Number of lesions			0.069	0.793
<3	52	51		
≥3	8	9		
KPS scores (points)	71.98 ± 3.65	72.15 ± 3.54	0.259	0.796
Marital status			0.046	0.831
Married	45	46		
Unmarried/divorced/widowed	15	14		
Educational degree				
Senior high school and below	25	26	0.034	0.853
Junior college	18	19	0.039	0.843
College and above	17	15	0.171	0.680

**Table 2:** Comparison of patients' short-term efficacy (*n* (%)).

Group	CR	PR	SD	PD	ORR	DCR
Experimental	18 (30.0)	30 (50.0)	6 (10.0)	6 (10.0)	48 (80.0)	54 (90.0)
Control	10 (16.7)	18 (30.0)	17 (28.3)	15 (15.0)	28 (46.7)	45 (75.0)
*X* ^2^	2.981	5.000	6.508	4.675	14.354	4.675
*P*	0.084	0.025	0.011	0.031	<0.001	0.031

## Data Availability

The data used to support the findings of this study are available on reasonable request from the corresponding author.

## References

[1] Lucinda B., Manning H., Emma C., Izzo L., Strockyj S. (2021). Thrombotic microangiopathy following a minor gynaecological procedure in the setting of endometrial cancer: a case report. *Case Reports in Women’s Health*.

[2] Tortorella L., Giovanni S., Francesco F. (2021). Author’s reply to: what is the prognostic importance of lymphovascular space invasion in the absence of lymph node metastasis for early-stage endometrial cancer?. *Journal of Gynecologic Oncology*.

[3] Aslan K., Mehmet Mutlu M. (2021). What is the prognostic importance of lymphovascular space invasion in the absence of lymph node metastasis for early-stage endometrial cancer?. *Journal of Gynecologic oncology*.

[4] Cem O., Sari Sezin Y., Guler Y. (2021). Outcome and safety analysis of endometrial cancer patients treated with postoperative 3D-conformal radiotherapy or intensity modulated radiotherapy. *Acta Oncologica*.

[5] Stefanos F., Nikolaos K., Anna G. (2021). The expression of NRIP1 and LCOR in endometrioid endometrial cancer. *In vivo (Athens, Greece)*.

[6] Zhao J., Hu Y., Zhao Y., Chen D., Fang T., Ding M. (2021). Risk factors of endometrial cancer in patients with endometrial hyperplasia: implication for clinical treatments. *BMC Women’s Health*.

[7] Ana O., Tinker Anna V., Gilbert L. (2020). Clinical activity and safety of the anti-PD-1 monoclonal antibody dostarlimab for patients with recurrent or advanced dMMR endometrial cancer: a nonrandomized phase 1 clinical trial. *JAMA Oncology*.

[8] Li J., Liu R., Tang S. (2019). Impact of endometriosis on risk of ovarian, endometrial and cervical cancers: a meta-analysis. *Archives of Gynecology and Obstetrics*.

[9] de Jonge Marthe M., de Kroon Cornelis D., Jenner Denise J. (2021). Endometrial cancer risk in women with germline BRCA1 or BRCA2 mutations: multicenter cohort study. *Journal of the National Cancer Institute*.

[10] Regina Esi Mensimah B. A., Daniela A., Sandra T., Amant F. (2021). Endometrial cancer molecular characterization: the key to identifying high-risk patients and defining guidelines for clinical decision-making?. *Cancers*.

[11] Goodman A. (2021). Is obesity predictive of endometrial cancer for women with postmenopausal bleeding?. *Menopause*.

[12] Wang B., Wang Q., Shi Y. (2021). Evaluation of clinical features related to lymphatic metastasis in grade 3 endometrial cancer: a retrospective cross-sectional study. *Chinese Medical Journal*.

[13] World Medical Association (2013). World Medical Association declaration of Helsinki: ethical principles for medical research involving human subjects. *The Journal of the American Medical Association*.

[14] Harada M., Yutaka O. (2021). Does polycystic ovary syndrome independently affect oncologic and reproductive outcomes in patients with endometrial cancer receiving fertility-sparing treatment?. *Journal of Gynecologic Oncology*.

[15] King M. T., Norman R., Mercieca-Bebber R. (2021). The functional assessment of cancer therapy eight dimension (FACT-8D), a multi-attribute utility instrument derived from the cancer-specific FACT-general (FACT-G) quality of life questionnaire: development and Australian value set. *Value in Health*.

[16] Visser Nicole C. M., van der Wurff Anneke A. M., Joanna I. H. (2021). Improving preoperative diagnosis in endometrial cancer using systematic morphological assessment and a small immunohistochemical panel. *Human Pathology*.

[17] Ha H., Chang H. K., Park S. J., Lim J., Won Y.-J., Lim M. C. (2021). The incidence and survival of cervical, ovarian, and endometrial cancer in Korea, 1999-2017: Korea Central Cancer Registry. *Obstetrics & Gynecology Science*.

[18] Lai Y.‐L., Chiang C.‐J., Chen Y.‐L. (2021). Increased risk of second primary malignancies among endometrial cancer survivors receiving surgery alone: a population‐based analysis. *Cancer Medicine*.

[19] Skorupa A., Poński M., Ciszek M. (2021). Grading of endometrial cancer using 1H HR-MAS NMR-based metabolomics. *Scientific Reports*.

[20] Jan B., Hanna R., Miłosz W. (2021). Association of Single Nucleotide Polymorphism LEP-R c.668A>G (p.Gln223Arg, rs1137101) of leptin receptor gene with endometrial cancer. *BMC Cancer*.

[21] Tang W., Kumaraguruparan R., Pillai Sureshkumar M. A. (2021). LIF/LIFR oncogenic signaling is a novel therapeutic target in endometrial cancer. *Cell Death Discovery*.

[22] Sofer A., Magnezi R., Eitan R. (2020). Robotic vs. open surgery in obese women with low-grade endometrial cancer: comparison of costs and quality of life measures. *Israel Journal of Health Policy Research*.

[23] Marcin L., Marcin Ś., Ewa W. (2021). Ultrasound measurement of tumor-free distance from the serosal surface as the alternative to measuring the depth of myometrial invasion in predicting lymph node metastases in endometrial cancer. *Diagnostics*.

[24] David F., Barbero Mark L., Werner Henrica M. J. (2021). Longitudinal effects of adjuvant chemotherapy and lymph node staging on patient-reported outcomes in endometrial cancer survivors: a prospective cohort study. *American Journal of Obstetrics and Gynecology*.

[25] Otter S., Stewart A. (2021). Cervical and endometrial cancer-a tale of two halves?. *Clinical Oncology*.

